# Autistic Adults' Experiences of Camouflaging and Its Perceived Impact on Mental Health

**DOI:** 10.1089/aut.2020.0071

**Published:** 2021-12-07

**Authors:** Louise Bradley, Rebecca Shaw, Simon Baron-Cohen, Sarah Cassidy

**Affiliations:** ^1^ International Centre, University of Bedfordshire, Luton, United Kingdom.; ^2^ Coventry and Warwickshire Partnership Trust, Coventry, United Kingdom.; ^3^ Autism Research Centre, University of Cambridge, Cambridge, United Kingdom.; ^4^ Cambridge Lifetime Asperger Syndrome Service (CLASS), Cambridgeshire and Peterborough NHS Foundation Trust, Cambridge, United Kingdom.; ^5^ School of Psychology, University of Nottingham, Nottingham, United Kingdom.

**Keywords:** autism, experiences, camouflaging, masking, mental health, qualitative research

## Abstract

***Background:*** Camouflaging (also referred to as “masking”) is a commonly reported strategy used by autistic adults in everyday life to help them cope in social situations. Autistic adults report that camouflaging can have a devastating effect on mental health and well-being, yet little is known about the lived experiences of camouflaging and its impact.

***Methods:*** We designed an online survey in partnership with autistic adults, to explore the experiences of camouflaging and its impact on mental health. Participants self-reported the lifetime experience of camouflaging, where they camouflaged the frequency and length of time spent camouflaging. Four open questions allowed participants to elaborate their answers to the closed questions on frequency and length of time, and subsequently any positive and negative aspects of their experience of camouflaging. Two hundred seventy-seven autistic adults who self-reported a diagnosis of an autism spectrum condition (128 female, 78 male) or self-identified as autistic (56 female, 15 male) were included in the analysis of qualitative responses to the open-ended questions.

***Findings:*** We thematically analyzed participant answers from the open questions. Three main themes emerged. First, “dangers of camouflaging” described how the amount of time spent camouflaging led to exhaustion, isolation, poor mental and physical health, loss of identity and acceptance of self, others' unreal perceptions and expectations, and delayed diagnosis. Second, “positive aspects of camouflaging” included greater access to social spaces, and protection from harm. Camouflaging was, therefore, seen as necessary to survive in a world designed for the neurotypical majority. Third, autistic adults described being diagnosed and accepted for who they are as reasons for “why I don't need to camouflage like I used to.”

***Conclusions:*** Time spent camouflaging is what seems to be most damaging for the participants' mental health. The main reason reported for needing to spend so much time camouflaging is society's lack of awareness and acceptance of autism.

**Lay summary:**

**Why is this study being done?**

Many autistic adults report that they need to camouflage their autistic behaviors to help them “fit in” and cope in social situations with non-autistic people. This is because society is not as aware and accepting of autistic people as it needs to be. We also know that for most autistic adults camouflaging is exhausting and damaging for their mental health. This study is important, because researchers have not studied camouflaging enough to know what it is like for autistic adults to camouflage in their everyday lives and to understand the impact that camouflaging has on their mental health.

**What was the purpose of this study?**

We wanted to ask autistic adults about their positive and negative experiences of camouflaging. This is important because it will help professionals better understand why autistic adults camouflage, and better support the mental health needs of autistic adults. This increased understanding may also help society become more aware and accepting of autism. If this happens, autistic adults will not need to camouflage as much. Not having to camouflage as much could also help prevent and reduce mental health problems in autistic adults.

**What did we do?**

We asked autistic adults with a clinical diagnosis and those who self-identify as autistic to complete an online survey. The survey asked questions about mental health, self-injury, suicidal thoughts, and suicidal behaviors. One part of the survey asked questions about camouflaging. If research participants said they camouflaged or masked their autistic characteristics to cope with social situations, they would then be asked about when and why they camouflage, and about the positive and negative consequences of camouflaging.

**What did we find?**

We found that autistic people confirmed that they camouflage because of a lack of awareness and acceptance of autism in society. We also found that both autistic males and females camouflage. Although some autistic adults said that “everyone” camouflages, they thought that autistic people spent much more time than non-autistic people camouflaging in their everyday lives. Spending lots of time camouflaging was what was most damaging for autistic adults' mental health. Although most autistic adults thought that camouflaging was damaging to their mental health, some thought that it helped them too.

**How will knowing this help autistic adults?**

Our results suggest that it is important to reduce pressure to camouflage. This could help prevent high rates of mental health problems in autistic people. Our results suggest that this can be achieved if wider society becomes more aware and accepting of autistic people. Our results also suggest that reducing pressure to camouflage could benefit everyone in society.

## Introduction

Up to 79% of autistic adults meet diagnostic criteria for a co-occurring psychiatric condition,^1^ and up to 66% reported feeling suicidal at some point in their life.^2^ However, there is a lack of research into *why* autistic people experience these high rates of mental health problems and suicidality.^3–5^ Previous research has suggested that autistic people, being the minority in a society designed for the neurotypical majority, attempt to camouflage, mask, or compensate for their autistic characteristics and behaviors, to “fit in” and access social spaces (e.g., relationships, work, education etc.).^6–11^ However, qualitative research has shown that autistic adults describe camouflaging as highly stressful, exhausting, and anxiety provoking.^6,7^ Camouflaging is also associated with feelings of not being accepted^12^ or belonging in society,^13–15^ which, in turn, increases risk of depression,^12^ suicidal thoughts and behaviors.^13–16^ In addition to increased risk of mental health problems and suicidality, camouflaging has also been argued to delay accurate and timely diagnosis of autism in adulthood, particularly among women,^17–19^ and prevent autistic adults' access to treatment and support for mental health problems.^20^

It has been argued that camouflaging is not a coping strategy used specifically by autistic people, but one of a range of potential coping strategies used in social situations, and it is present in a number of different groups.^21^ The tendency to camouflage one's autistic traits has been found to be normally distributed in both autistic adults and the general population,^9,10^ and associated with poor mental health and suicidality in both autistic and non-autistic people.^10,15^

Gender differences in camouflaging have also been explored, but the findings are inconsistent. Some studies suggest that camouflaging may be more prevalent in autistic females than autistic males and may explain why autistic females tend to be under- or misdiagnosed,^17–19,22–24^ but this has not been replicated in all studies.^7,8^ A further study found no significant difference between rates of self-reported camouflaging between autistic males (89.2%) and autistic females (90.9%). However, autistic females reported camouflaging in significantly more situations and for more of the time than autistic males,^16^ suggesting that there are more subtle and nuanced gender differences than camouflaging being female-specific.

Associating camouflaging with a “female specific” presentation of autism could lead to disparities in diagnosis with nonbinary people and males not being diagnosed, because they do not fit the “female phenotype.”^25^ It is crucial that we better understand autistic adults' experiences of camouflaging (regardless of gender), the impact of camouflaging on mental health, and how this impact could be reduced. The current study, therefore, aimed to qualitatively explore autistic adults' experiences of camouflaging and the impact on their mental health. Many autistic adults remain undiagnosed, which may, in part, be due to successful camouflaging.^17–19^ We, therefore, recruited both autistic adults who reported having a confirmed diagnosis and those who self-identified as autistic.

## Methods

### Design

This study uses data from a large online survey about mental health in autistic adults.^16,20,26^ Given that autistic people's voices have been excluded from much research about them,^27^ we co-produced this large online survey in partnership with a steering group of eight autistic adults, to explore their experiences of mental health problems, self-injury, and suicidality. We asked the steering group what aspects of the project they would like to work on with us and their decision was to be involved in the design, recruitment, and dissemination stages of the project, particularly the survey, rather than analysis or write-up.^1^ The group advised us that an online survey would be the most appropriate method for collecting data, as it would allow autistic individuals to participate in familiar surroundings, complete the survey in their own time, and capture a much larger range of experiences than would have been possible with face-to-face interviews.

The autistic steering group prioritized the topics to be covered across a number of sections in the survey. These sections included: demographics, diagnoses, camouflaging, autistic traits, non-suicidal self-injury, suicidality, treatment and support, and suggestions for future suicide prevention efforts. Given the sensitive topics in the survey, participants could skip sections that they did not want to complete. Participant numbers, therefore, differed slightly for each section of the survey. Data from the section of the survey exploring autistic adults' experiences of camouflaging are presented here. Previous studies present data from other sections of the survey.^16,20,26^

We recruited participants to the survey from the research volunteer's database located in the Autism Research Centre at the University of Cambridge. Autistic adults and their family members across the United Kingdom internationally registered in the Cambridge Autism Research Database (CARD). In addition, participants were recruited from online adverts. We invited autistic (diagnosed and self-identifying) adults (18 years and older) to complete an online survey about understanding and preventing mental health problems, self-injury, and suicidality. Participants could take part with or without previous experience of mental health problems, self-injury, and suicidality.

### Participants

Three hundred forty-six autistic adults (251 diagnosed, 95 self-identifying) consented to take part in the wider survey about mental health in autistic adults. Of these, 334 autistic adults (242 diagnosed, 91 self-identifying) started the camouflaging section, with 300 of these (89.8%) (219 diagnosed, 81 self-identifying) endorsing life-time experience of camouflaging. Of these, 277 (128 diagnosed, 56 self-identifying) subsequently provided qualitative data included in the current study (see Table 1 for participant demographics of this subsample). All 277 participants answered “Yes” to the question, “Have you ever tried to camouflage or mask your ASC characteristics to cope with social situations” and responded to at least one open text question (see below for further details of the camouflaging questionnaire).

**Table 1.** Participant Demographics Table

Variables	Group			
	ASC(D) male* *(*n* = 78)	ASC(D) female* *(*n* = 128)	ASC(SI) male* *(*n* = 15)	ASC(SI) female* *(*n* = 56)
	Mean (SD)/*n *(%)			
Age	42 (11.65)	36.42 (10.57)	36.57 (9.83)	39.34 (8.64)
AQ total score	35.48 (7)	37.62 (7.75)	32.8 (7.82)	34.29 (6.28)
Age diagnosed with ASC	36.65 (13.93)	32.31 (12.04)	—	—
Camouflage total score	13.12 (3.99)	14.9 (3.6)	10.93 (4.7)	13.34 (3.61)
Lifetime NSSI	41 (56.9)	85 (78.7)	6 (42.9)	43 (81.1)
Suicidality				
SBQ-R total score	10.49 (4.13)	10.88 (3.96)	10.21 (3.77)	9.56 (3.89)
*% ≥ general population cutoff*	60 (80)	96 (82.1)	11 (78.6)	38 (73.1)
*% ≥ psychiatric population cutoff*	55 (73.3)	90 (76.9)	11 (78.6)	35 (67.3)
Lifetime suicidal ideation	19 (25.3)	14 (12)	3 (21.4)	10 (18.9)
Lifetime suicide plan	27 (36)	46 (39.3)	7 (50)	24 (45.3)
Lifetime suicide attempt	26 (34.7)	51 (43.6)	3 (21.4)	25 (26.4)
ASC subtype				
HFA/AS	63 (80.8)	113 (88.3)	—	—
Autism/classic autism	0 (0)	3 (2.3)	—	—
ASC unspecified	9 (11.5)	7 (5.5)	—	—
PDD/PDD-NOS	1 (1.3)	1 (0.8)	—	—
Other	5 (6.4)	4 (3.1)	—	—
Education type				
Mainstream	70 (89.7)	113 (88.3)	15 (100)	54 (96.4)
Home	1 (1.3)	3 (2.3)	0 (0)	1 (1.8)
Special	3 (3.8)	6 (4.7)	0 (0)	0 (0)
Private/boarding	4 (5.1)	6 (4.7)	0 (0)	1 (1.8)
Support				
Need/receive support	60 (78.9)	95 (79.8)	5 (33.3)	37 (71.2)
Unmet support needs^a^	3.03 (2.65)	3.67 (2.61)	2.8 (2.77)	3.05 (2.34)
Occupational status				
Employed	36 (46.2)	67 (52.8)	10 (71.4)	34 (61.8)
Volunteering	5 (6.4)	9 (7.1)	0 (0)	2 (3.6)
Student	6 (7.7)	24 (18.9)	1 (7.1)	1 (1.8)
Unemployed/unable to work	29 (37.2)	25 (19.7)	3 (21.4)	17 (30.9)
Retired	2 (2.6)	2 (1.6)	0 (0)	1 (1.8)
Mental health or other condition				
≥1 mental health or other condition	67 (85.9)	117 (92.1)	9 (64.3)	44 (78.6)
Depression	60 (76.9)	103 (81.1)	8 (57.1)	40 (71.4)
Anxiety	49 (62.8)	95 (74.8)	6 (42.9)	35 (62.5)
Obsessive compulsive disorder	11 (14.1)	28 (22)	0 (0)	5 (8.9)
Bipolar disorder	4 (5.1)	11 (8.7)	0 (0)	4 (7.1)
Personality disorder	6 (7.7)	19 (15)	1 (7.1)	6 (10.7)
Schizophrenia	3 (3.8)	5 (3.9)	0 (0)	0 (0)
Anorexia nervosa	1 (1.3)	12 (9.4)	0 (0)	6 (10.7)
Bulimia	0 (0)	4 (3.1)	0 (0)	4 (7.1)
Myalgic encephalopathy	4 (5.1)	11 (8.7)	1 (7.1)	6 (10.7)
Tourettes	2 (2.6)	3 (2.4)	0 (0)	0 (0)
Epilepsy	2 (2.6)	4 (3.1)	1 (7.1)	3 (5.4)
Other	13 (16.7)	26 (20.5)	0 (0)	11 (19.6)
Developmental condition				
≥1 developmental condition	15 (19.2)	32 (25)	1 (7.1)	42 (75)
Dyspraxia	4 (5.1)	15 (11.7)	0 (0)	4 (7.1)
Learning disability	2 (2.6)	0 (0)	0 (0)	1 (1.8)
Learning difficulty	0 (0)	2 (1.6)	0 (0)	2 (3.6)
Dyscalculia	3 (3.8)	3 (2.3)	0 (0)	2 (3.6)
Dyslexia	6 (7.7)	10 (7.8)	0 (0)	8 (14.3)
Attention deficit hyperactivity disorder	2 (2.6)	14 (10.9)	1 (7.1)	3 (5.4)
Developmental delay	1 (1.3)	2 (1.6)	0 (0)	1 (1.8)
Other	3 (3.8)	5 (3.9)	0 (0)	1 (1.8)

^a^ represents unmet support needs calculated by (total n areas support ideally liked—total n areas support actually received).

AQ, autism spectrum quotient; AS, asperger syndrome; ASC, autism spectrum condition; D, diagnosed; HFA, high functioning autism; NSSI, non-suicidal self-injury; PDD, pervasive developmental disorder; PDD-NOS, pervasive developmental disorder - not otherwise specified; SBQ-R, suicidal behaviours questionnaire - revised; SD, standard deviation; SI, self-identified.

### Camouflaging survey questions

We included a section on camouflaging in the online survey, which comprised four closed and four open-ended questions. First, we asked participants a screening question: “Have you ever tried to camouflage or mask your characteristics of ASC to cope with social situations? For example, have you ever tried to copy or mimic other people's behaviour to try and fit in (e.g., copying another person's accent or mannerisms), or tried to mask or hide your characteristics of ASC from other people?” If they answered “yes,” we asked participants to specify the areas in which they camouflaged (work, educational settings, social gatherings, when visiting the doctors, when visiting a health professional, at home, with friends, other), and the overall frequency they camouflaged on a scale from 1 (never) to 6 (always [over 90% of social situations]). We then asked participants, “If you wish, please use this space to explain your answer” and provided an essay text box without character or word limit to answer.

We also asked participants to specify the overall amount of the day that they spend camouflaging on a scale from 1 (none of my waking time) to 6 (all of my waking time (over 90% of social situations)). Again, we asked participants, “If you wish, please use this space to explain your answer” and provided an essay text box to answer. We then asked participants further two open-ended questions: “What have been the positive consequences (if any) of camouflaging your autism?” and “What have been the negative consequences (if any) of camouflaging your autism?” Both questions were followed by essay text boxes.

### Data analysis

We conducted a thematic analysis, using a data-driven inductive approach, at a semantic level, following the Braun and Clarke framework.^28,29^ We chose this method, as it is ideal to identify patterns of information from qualitative data gathered through online survey methods.^30,31^ Further, we chose a data-driven inductive approach to enable the identification of new perspectives and information relating to a relatively underexplored topic (experiences of camouflaging and the impact on mental health), rather than using a rigid theoretical framework for interpretation. Although thematic analysis is recognized as an active process, where researchers' experience naturally influences the themes to some extent, we conducted the analysis from an essentialist theoretical position with experiences understood as the participants' reality. L.B. read and familiarized themselves with the data and generated initial codes. L.B. then grouped codes describing similar concepts into initial themes. L.B. and S.C. met regularly to discuss and review the analysis to develop the final themes.

We present quotes from the survey verbatim and referenced with participant numbers. We prefaced participant quotes with M (male) or F (female) and followed with D (diagnosed) or SI (self-identified) to provide additional information for the reader, for example, “M235-D” or “F456-SI.” As the dataset was large, when participant experiences were shared, we provided the number of similar responses. We counted responses after thematic analysis was complete and so the numbers are not an analytic focus but provided as an additional layer of information for the reader.^20,30,31^ These numbers are not a measure of significance but provided to give the reader a sense of how much an experience was shared across the dataset.^20,30,31^ We have changed quotes throughout where pronoun disagreement could be confusing, symbolized with square brackets. For example, we changed “I” to “[she]” or we changed “[he]” and “my” to “[their].” We received ethical approval for the study from our local research ethics committee. Our study was also approved by our autistic steering group who co-designed the study, and the scientific advisory group at the Autism Research Centre, University of Cambridge, prior to recruiting participants registered in the Cambridge Autism Research Database (CARD).

## Findings

Participants described their experiences of camouflaging as the need to “adapt behaviour to appear more socially acceptable” (M759-D), describing how they spend time “watching others” (F199-D), “mimicking social behaviours” (F436-D), or attaching themselves to someone more social “to give the illusion of being sociable” (F72-SI). They describe adopting a set of “rules” (F272-SI), “social mode” (M630-D), “people suit” (M438-D), or “invisible mask” (F485-D) that they would use to cope with everyday social situations as if playing a “game” (F140-SI), “part” (F140-SI), or “role” (F178-D). Individuals reported having camouflaged so much their whole life that it “became second nature” (F193-D). They described it as being “hard to now turn off” (F483-D), because camouflaging “is my default” (F238-D), something that is done “subconsciously” (M81-D), “without thinking” (F127-D), as if on “autopilot” (M100-D).

**FIG. 1.**
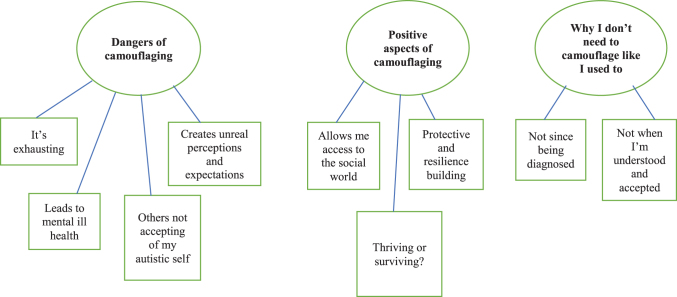
Thematic map.

Three themes emerged when analyzing the data that described the participants' experiences and impact of camouflaging (Fig. 1).

### Theme 1: dangers of camouflaging

Although the survey did not ask questions related to camouflaging and gender differences, participants made reference to camouflaging being something that they thought “everyone” did (six participants). However, it was the amount of *time* that autistic people spent camouflaging and the impact this had that seemed to be autism-specific and reported as being “really damaging” (F224-D):

“I think that everyone, neurotypical or not, camouflage their real selves in one way or another…But, for autistics, we have to camouflage all the time, even with people we know pretty well, or our families, and the…consequences associated with it are much more deeply felt by autistics than neurotypicals” (F758-SI).

Participants reporting to “always” camouflage (79 participants) described needing to “constantly monitor” (M202-D) their behavior, so much so that they did not know “how to be any different any more” (F18-D), or how to “exist in social situations without camouflaging” (F21-D).

#### It's exhausting

One of the most commonly shared narratives that came out of the data for this theme was how “exhausting” (73 participants), “tiring” (33 participants), or “stressful” (44 participants) camouflaging was. Participants explained there being “so many rules of what to do in each situation” (F477-D), that their brain was described as “constantly working and ticking” (F99-D), “building patterns and sub-patterns to describe every situation based on every person” (M632-D). These “rules” were needed to help participants get “all of the social cues right” (F136-D) “have the right facial expression” (F39-D) and respond in the “right way” (F39-D):

“It is EXHAUSTING. It's like trying to solve mathematical equations in your head all day long while carrying on as normal” (F126-D).

Many described being exhausted after social interactions because of the effort it took to “pretend” (27 participants) they are “neurotypical” (23 participants), “normal” (57 participants), or “don't have ASC^2^” (23 participants). This left them feeling “cognitively overloaded” (F9-D), “overwhelmed”, (F55-D) or “burnt-out” (F10-D). Therefore, this made the “seemingly simple things” (F18-D) such as “eating or washing or brushing hair” (F35-D) difficult because camouflaging took all of their energy:

“Its the most debilitating part of my ASD^3^… I can't stress enough how massive this is and the difficulties faced by myself and everyone around me because of my ability to blend in over the years… I despise how being good at camouflaging has impacted life across the board” (F118-D).

The exhausting nature of camouflaging meant that participants needed time to “recover” (F30-D) and “recharge” (M630-D). If participants were unable to take recovery days, they described not bring able to “cope” (4 participants), putting them at risk of a “meltdown” (10 participants). Individuals explained how they would, “shut down several days a week” (F653-D), or not “leave the house” (M799-D) after “stressful and uncomfortable” (F29-D) social situations. Further, participants described experiencing physical ill health (18 participants) such as “headaches” (6 participants), “gut issues” (F89-D), and “aching muscles” (F11-D) after camouflaging. One participant explained that camouflaging “feels almost painful” (F483-D).

#### Leads to mental ill health

Although camouflaging was reported to impact physical health, it was the participants' “mental health” (72 participants) that seemed to suffer the greatest, with “depression” (23 participants) and “anxiety” (44 participants) being most reported:

“I didn't realise how badly my Autism affects my anxiety, and people do not realise how much I suffer when trying to camouflage…Its a horrible cycle of poor mental health and I feel like I could prevent it if only others would accept my diagnosis” (F361-D).

Participants reported that camouflaging made them more likely to have “mental health breakdowns” (four participants), “suicidal thoughts” (five participants), or to “self-injure” (three participants) due to the “pressure” (F23-D) of needing to cope in social situations. One participant explained that her mental ill health was “a direct consequence of social rejection when [she] couldn't mask adequately” (F9-D). However, others described using alcohol (five participants) to get “through social events” (three participants), as it helped them “feel less anxious and self aware” (F7-D).

#### Others not accepting of my autistic self

Camouflaging to “fit in” (50 participants), avoid being “bullied” (15 participants), or “abused” (9 participants) was reported by many who expressed a desire to be “accepted” (43 participants) without needing to camouflage their autistic characteristics. Individuals described, “being me is never acceptable I'm a social lepper” (F161-D), and that “it's really not possible to be ‘out’ and natural all the time without incurring stigma and disapproval” (F244-D) because “people sense ‘different’ and withdraw from it” (M614-D):

“I in myself feel as if society is rejecting me for who I am. Some say you should be yourself. How can anyone say that when society's expectations do not match with yourself? When that time comes, you have no choice but to change, so you're forced to struggle against a delicate balance between conformity and individuality” (M753-D).

Consequently, many reported isolating themselves to “avoid social situations” and “spending time with people” (16 participants) because of difficult past experiences:

“People react very badly to me. I upset them constantly. I am devastated by this…so I stay home alone whenever I can, so I don't upset people” (F478-D).

Many described having lost the “real me” (15 participants), or feeling “fake” (17 participants), which affected their “confidence,” “self-worth,” “self-esteem,” and “identity” (21 Participants). Participants reported not knowing “what this concept of ‘self’ really is” (M636-D), and having “little of ‘me’ left” (F610-D) because they were “a patchwork of acts” (M196-D), leading to a sense of “shame” (five participants):

“[I] often hate myself because of not being publicly acceptable as who I really am” (F134-D).

#### Creates unreal perceptions and expectations

Participants reported that camouflaging made it difficult for others to get a “measure” (F133-D) of them, causing “confusion” (M435-D), “disbelief” (F161), and people to be “astounded” (F89-D) when told they are autistic. Individuals described receiving comments such as, “you don't look autistic” (F483-D), you “look normal” (F608-D) or seem “fine” (M657-D) because autism “is not visually evident” (F139-D). This resulted in some participants feeling there was not enough “recognition of [their] difficulties” (M438-D), that people “expect too much” (F483-D), and “think [they] are coping when [they] are not” (M673-D):

“People think I manage my life better than I actually do. Unfortunately this means that people don't think I need the help that I sometimes do, or they think ‘You're capable, why can't you do that?’” (F189-D).

Participants explained that camouflaging does not always work and that it is “easy to slip up and make a mistake” (F123-D). Therefore, “when the mask slips” (12 participants), “the consequences are massive” (F21-D) as the “real me” (M657-D) “is extreme in comparison to the fake me” (F21-D), and people think “I'm being intentionally rude or arrogant” (F61-D), or they are “offended” (M438-D), “shocked and reject me” (F478-D).

Further, camouflaging was reported to cause a “delay in formal diagnosis” (M438-D), which for one participant “probably directly precipitated the extended mental health crisis” (M438-D) he experienced. Others explained that, “an earlier diagnosis would have been a major benefit in [their] younger years” (M438-D), and meant they “wouldn't have hurt people and [themselves] to the same extent” (F118-D).

### Theme 2: positive aspects of camouflaging

Although more damaging experiences of camouflaging were reported, many described reasons why they camouflaged and what it afforded them when asked if there were any positives to camouflaging.

#### Allows me access to the social world

One of the most commonly shared narratives that came out of the data for this theme was that it allowed participants “access to advantages of the neurotypical world” (F683-D). Many described how camouflaging helped them feel “normal” and “accepted” (48 participants), or able to “function” and “get through life” (14 participants). Others described how it helped them “interact” and “meet people” (23 participants), find “work” (37 participants), “friendships” (20 participants), “partners” (5 participants), and have “children” (6 participants):

“…it enables me to function in the world…hold down a job, be a parent, travel on the bus and train, go shopping. I can do ‘normal’ things” (F378-D).

#### Protective and resilience building

For some participants, the “safety” (F756-D) of camouflaging enabled them to “cope” (M626-D) and feel “protected” (F181-D), more “confident” (F96-D), and “capable of being in the world” (F588-D):

“I grew up in a time when the diagnostic criteria for autism were tighter, so I would have never been diagnosed as autistic as a child…‘acting normal’ was a protective factor when I was seriously depressed and kept me out of NHS secondary mental health care at a time when my mental health problems would have been treated in a way that would have damaged my education and probably hospitalised me” (F342-D).

The concept of resilience seems to capture the narrative described by many: “it makes me work harder than others…to compensate for what I don't naturally possess” (F144-D), “I can achieve what I want to achieve…in spite of any obstacles my ASD may present” (F127-D). Others reported that camouflaging helped them think about their hopes, so they were able to “regain [their] life” (M619-D) and “have a ‘future’ of whatever that maybe despite of difficulties” (F251-D):

“I do feel a sense of achievement as I have had what many would see as a successful career, albeit that it was a huge struggle at times. No allowances were ever made for my condition…I therefore feel I have overcome many obstacles others have not had to” (M259-D)

#### Thriving or surviving?

Although many participants reported positive aspects of camouflaging, these reported benefits prompted us to ask the question—is this thriving or surviving? Participants described camouflaging as enabling them to “survive” (four participants), “not stand out” (seven participants), and be “bullied” (seven participants). When camouflaging, they described there being “less chance of being discriminated against” (F11-D), “attacked” (M88-D), feeling like “a burden on society” (F118-D), “labelled” (F20-D), “judged” (F38-D), “targeted” (F156-D), or “abused” (F59-D). Individuals explained that when camouflaging they, “don't get rocks thrown at [them] for being weird” (F78-D), and that others do not “know [they're] different and won't try to kill [them] for…causing them inconvenience” (F153-D):

“Not everyone is willing to care enough about you to learn that the quirks have a good side. When they see someone in a wheelchair, they know what to expect, but we have no visible cues, so they expect neurotypical behaviour. When the interactions are short and meaningless, camouflaging is worth doing as it won't increase the stress too much and you probably won't see those people again” (M632-D).

Others not knowing the participants were “autistic” (F365-D) was reported to be a positive (10 participants) as it meant they could pass as being “normal” (16 participants), making others “feel comfortable” (M810-D), and “not afraid to approach and be around me” (F105-D).

These experiences seem to present survival rather than thriving, highlighting the expectation placed on autistic people to adapt to have access to the social world and therefore society's lack of awareness and understanding.

### Theme 3: why I don't need to camouflage like I used to

#### Not since being diagnosed

One of the most commonly shared narratives that come out of the data for this theme was that many participants reported not camouflaging like they used to after being given a “diagnosis” (17 participants) of autism:

“Since receiving my diagnosis, I am consciously not camouflaging. On the contrary, I make it known that I'm on the Autistic Spectrum and explain why any particular situation is stressful or uncomfortable. I do this to help my own needs and to educate others about ASC” (F29-D).

Participants who camouflaged less felt more “positive” (F301-D), “accepted” (two participants), and “confident” (F240-D), as they were not wanting to “hide [their] autism” (F9-D). However, for others wanting to camouflage less, the change seemed more difficult:

“I still feel quite ashamed of myself…because of the stigmatising attitudes I hear from people around me…I am getting slowly better at being comfortable not putting [on] my armour, around people I'm reasonably comfortable with, and finally getting a diagnosis has played a key role in that” (M438-D).

Others reported to camouflage less than they used to, because they just found it too “exhausting” (M657-D) and “extremely tiring” (F25-D) because it “takes too much energy to try and hide” (F37-D):

“It is hugely stressful and exhausting and, until I finally accepted that I was on the spectrum it left me feeling isolated and alien because I just couldn't seem to get how to think in the ‘right’ way which caused me a lot of anxiety” (F159-D).

For others it was because they felt they were not “very good at it anyway” (three participants), or that they did not need to camouflage as much because “friends were accepting” (three participants) or because their “employer wants to make reasonable adjustments” (F342-D).

Some could not “see the point anymore” (M125-D), or questioned “why [they] should any more” (M420-D):

“It only benefits the ‘normal’ people around me, by allowing them to just pretend everything is fine and not have to give a single thought to communicating clearly…” (M657-D).

“[I'm] trying to view autism as more of a positive thing that I shouldn't have to hide but this is hard to learn when society isn't always supportive” (F301-D).

#### Not when I'm understood and accepted

Another commonly shared narrative that comes out of the data for this theme was that participants did not feel the need to camouflage as much, if at all, when they spent time with people who “understand” or “accept” autism (3 participants), and when they were with certain members of their “family and friends” (11 participants), their “partner” (10 participants), or other “autistic” or “neuordiverse” people (5 participants):

“I don't camouflage my autism when I'm with my dad (who accepts me and my quirks fully) or with my autistic friend (whom I met on the Internet and recently met for the first time in person. It is SO UNBELIEVABLY WONDERFUL to be able to spend time with someone without masking!!). All of the rest of the time I either have to camouflage completely (e.g., at university) or partially (e.g., with my mum, who tells me off if I'm too weird)” (F5-D).

One participant described that the benefit of not camouflaging was felt not only by themselves but also by others around them:

“After I received the diagnosis I decided to stop camouflaging altogether, to see what was the Aspergers and what was just myself. My wife said “I got much worse” as if I “gave up caring”, but I was actually just getting to know myself, someone I had no idea existed since very young. Now, my wife, friends and workplace got to know who I really am, and I think they're getting used to it and are learning to enjoy my different behaviour” (M632-D).

## Discussion

Many participants described the damaging impact of camouflaging: It was exhausting, isolating, damaging for their mental and physical health, identity, and acceptance of self, created unreal perceptions and expectations of their abilities for others, and in some cases led to a delay in formal diagnosis or a mental health crisis. Participants reported needing to camouflage to such an extent because of a lack of awareness, understanding, and acceptance of autism in society. These themes are largely consistent with previous qualitative studies of camouflaging in autistic adults^6,7^ and resonate with previous research that shows that lack of autism acceptance is a strong motivator for camouflaging but with consequent negative impacts on mental health.^12^

Although more damaging experiences of camouflaging were reported, many positives were described: It gave participants access to the social world, facilitated relationships, careers, protected them from harm, and helped them build resiliency and overcome challenges. Although these experiences were described as positives, they prompted us to ask the question: Is this thriving or surviving? This was especially when reference was made to how camouflaging helped some escape abuse, bullying, and enabled them to not feel like a “burden on society.” These “positives” of camouflaging appear to be necessary to survive a world built for the neurotypical majority. Therefore, the positives of camouflaging come from what it affords rather than the act of camouflaging itself, but this might come at too great a cost considering the dangers described in theme one.

It was the amount of *time* spent camouflaging that participants described as being the most damaging aspect, especially for their mental health. Camouflaging was reported to such an extent in some cases that participants described no longer being able to see how they could function without camouflaging. Crucially, however, is that if camouflaging is not considered autism-specific,^21^ everyone is likely to be able to relate to the experience of camouflaging on some level. This shared human experience could potentially help increase society's awareness, understanding, and acceptance of autism, neurodiversity, and differences, therefore reducing the need to camouflage benefiting the whole of society.^14^ This is vitally important, as those who reported not needing to camouflage like they used to describe the change positively and beneficial not only for themselves but also for others around them. Increasing society's awareness, understanding, and acceptance so autistic people would not have to camouflage as much would also have the potential to reduce the risk associated with poor mental health and suicidality, not only improving but also saving lives.^14–16^

Although we did not specifically ask about gender differences, none of the 277 participants made reference to camouflaging in terms of their gender. Both autistic men and women reported experiences of camouflaging, and their experiences of camouflaging did not appear qualitatively different. The danger of camouflaging being seen as a female only coping strategy is that clinicians may not recognize camouflaging in males.^25^ This means that any associated mental health needs related to camouflaging might be missed, or not treated or supported effectively. It is, therefore, vitally important for clinicians and professionals to better understand camouflaging in autistic *people*, and make these conversations part of the treatment, therapeutic, and support plans of those seeking help, regardless of gender. Further, training for clinicians in autism acceptance will also be important to help reduce pressure to camouflage in clinical contexts.

This study has a number of limitations and strengths. Participants self-reported their autism diagnostic status. However previous research has shown good agreement between self-reported and clinically confirmed diagnoses.^32^ Respondents to the online survey were autistic adults, largely diagnosed in adulthood, without co-occurring intellectual disability, and largely female—opposite to the gender bias found in the wider autistic population. Advertising the study was not topic blind—participants knew the survey would cover experiences of mental health, self-injury, and suicidality, including treatment and support, personality, and coping in social situations. The survey was only available online and there was no postal version available so only those with access to a computer and Internet could complete the survey. Our analysis of data was not double coded and did not include an autistic perspective. A key strength and novel aspect of the study was that we designed the survey and questions in partnership with autistic adults, who ensured that the content and design of the survey was accessible, ethical, and relevant. A further strength was that we collected the data via an online survey. This allowed us to collect a larger qualitative dataset than other methods (e.g., interviews) would allow.^30^ As so little is known about the lived experiences of camouflaging, and its impact, this article provides important insights to better understand camouflaging in autism for researchers, clinicians, and the autism community.

## Notes

^
1
^
This paper was written after the project ended and so the steering group was no longer convened and available to contribute.

^
2
^
Autism spectrum condition.

^
3
^
Autism spectrum disorder.
